# Bone marrow-derived mesenchymal stem cells promote colorectal cancer progression via CCR5

**DOI:** 10.1038/s41419-019-1508-2

**Published:** 2019-03-19

**Authors:** Gen Nishikawa, Kenji Kawada, Jun Nakagawa, Kosuke Toda, Ryotaro Ogawa, Susumu Inamoto, Rei Mizuno, Yoshiro Itatani, Yoshiharu Sakai

**Affiliations:** Department of Surgery, Graduate School of Medicine, Kyoto University, Kyoto, Japan

## Abstract

Mesenchymal stem cells (MSCs) are recruited from BM to the stroma of developing tumors, where they serve as critical components of the tumor microenvironment by secreting growth factors, cytokines, and chemokines. The role of MSCs in colorectal cancer (CRC) progression was controversial. In this study, we found that C-C chemokine receptor type 5 (CCR5) ligands (i.e., C-C motif chemokine ligand 3 (CCL3), CCL4, and CCL5) were highly produced from MSCs using a chemokine array screening with conditioned media from the cultured human MSCs. A relatively strong CCR5 expression could be detected within the cytoplasm of several CRC cell lines. Regarding the effect of MSC, we found that the xenografts in which CCR5-overexpressing HCT116 cells were inoculated into immunocompromised mice were highly promoted in vivo by a mixture with MSCs. Notably, the CCR5 inhibitor, maraviroc, significantly abolished the MSC-induced tumor growth in vivo. In human clinical specimens (*n* = 89), 20 cases (29%) were high for CCR5, whereas 69 cases (71%) were low. Statistical analyses indicated that CCR5 expression in primary CRC was associated with CRC patients’ prognosis. Especially, stage III/IV patients with CCR5-high CRCs exhibited a significantly poorer prognosis than those with CCR5-low CRCs. Furthermore, we investigated the effects of preoperative serum CCR5 ligands on patients’ prognosis (*n* = 114), and found that CRC patients with high serum levels of CCL3 and CCL4 exhibited a poorer prognosis compared to those with low levels of CCL3 and CCL4, while there was no association between CCL5 and prognosis. These results suggest that the inhibition of MSC–CRC interaction by a CCR5 inhibitor could provide the possibility of a novel therapeutic strategy for CRC, and that serum levels of CCL3 and CCL4 could be predictive biomarkers for the prognosis of CRC patients.

## Introduction

Colorectal cancer (CRC) is the third most common cancer and the fourth most common cause of cancer death worldwide, accounting for about 1.36 million deaths every year^1^. If it remains localized, the 5-year survival rate is about 90%. When it spreads to lymph nodes and distant organs, the 5-year survival rate reduces to about 70% and to about 10%, respectively^2^. There is mounting evidence that the tumor–stromal interaction, so-called tumor microenvironment (TME), promotes tumor invasion and metastasis through chemokine signaling^3^. The tumor stroma contains extracellular matrix and several types of host cells, including immune cells, vascular cells, and mesenchymal cells. The molecular mechanisms involved in the recruitment of these stromal cells to the tumor are poorly understood.

Mesenchymal stem cells (MSCs) were first discovered in bone marrow (BM) stroma in the 1960s^4^, and are pluripotent progenitor cells that contribute to the normal homeostasis and remodeling following injury in a variety of tissues^5^. Although MSCs reside predominantly in the BM, they can be distributed throughout the body. MSCs display some properties, such as the tendency to home into the sites of injury, capacity to suppress immune reactions, and promote the repair of damaged tissues. Due to these properties, MSCs have been explored for their application as cell sources in regenerative medicine and as delivery vehicles in gene therapy^5^. Growing tumors are considered as “chronic wounds that do not heal^6^”, and can activate the recruitment of host cells to promote proliferation and survival of tumor cells. In the context of cancer, recent studies have proposed that MSCs are recruited from BM to the stroma of developing tumors, where they serve as critical components of the TME. For example, Karnoub et al. reported that when breast cancer cells were mixed with MSCs and injected subcutaneously, lung metastasis was accelerated through the CCL5–CCR5 axis in the experimental mouse models^7^. Some chemokines and cytokines have been reported to be involved in the interaction between MSCs and cancers^8,9^.

In the present study, we investigated the interaction between CRC cells and MSCs, focusing on the chemokine signaling. We demonstrated that human MSCs secreted high levels of CCR5 ligands (i.e., CCL3, CCL4, and CCL5), and that MSCs promoted CRC tumor growth in vivo via CCR5 signaling. The xenografts in which CCR5-overexpressing CRC cells were inoculated into immunocompromised mice were highly promoted by a mixture with MSCs. Notably, we found that the CCR5 inhibitor, maraviroc, abolished the MSC-induced tumor growth in vivo. In human clinical specimens, we found that CCR5 expression in primary CRC was associated with CRC patients’ prognosis. Especially, stage III/IV patients with CCR5-high CRCs exhibited a significantly poorer prognosis than those with CCR5-low CRCs. We further showed that CRC patients with high serum levels of CCL3 and CCL4 exhibited a poorer prognosis compared to those with low levels of CCL3 and CCL4. These results suggest that a CCR5 inhibitor may provide the possibility of a novel therapeutic strategy for CRC, and that serum levels of CCL3 and CCL4 could be predictive biomarkers for the prognosis of CRC patients.

## Results

### CCL3/4/5–CCR5 axis is a candidate that functions between MSCs and CRC cells, which resulted in tumor growth in vivo

**Fig. 1:**
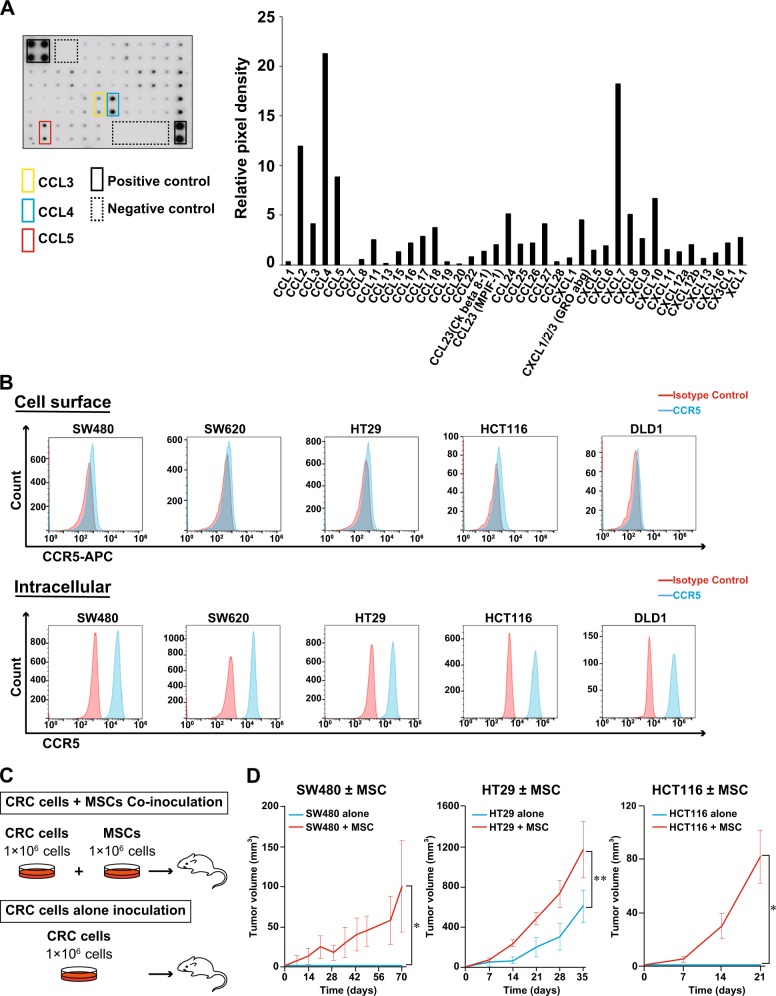
Relationship between colorectal cancer (CRC) cells and mesenchymal stem cells (MSCs). **a** Chemokine array using supernatants collected from MSCs. Array membrane (left) and relative intensity values of each chemokine (right). A positive control was set as 100. **b** Cell surface expression (top) and intracellular expression (bottom) of C-C chemokine receptor type 5 (CCR5) analyzed by flow cytometry. Red area represented isotype IgG control and blue area did anti-CCR5 Ab. **c** Schema of co-inoculation mice model. **d** Xenograft growth curves of SW480 (letf), HT29 (middle), and HCT116 (ritght) injected with or without MSCs. Mean; bar, ±SE, *n* = 4 (SW480), *n* = 6 (HT29 and HCT116) (Student’s *t* test; *P < 0.01, **P = 0.03)

Based on the previous reports that have shown that MSCs promote tumor progression in several types of cancer including breast cancer^7,10^, prostate cancer^11,12^, and gastric cancer^13–15^, we hypothesized that the chemokine crosstalk between MSCs and tumor cells played a role in the CRC microenvironment. To explore a candidate that functions between MSCs and CRC cells, conditioned media from the cultured human MSCs were screened for various chemokines using a chemokine array kit. Of note, several chemokines were produced by MSCs at a relatively high level, and, especially, CCL3, CCL4, and CCL5 were highly detected (Fig. 1a). We also confirmed the expressions of these chemokines at mRNA levels by quantitative reverse transcription-PCR (qRT-PCR; data not shown). Because the cognate receptor for these chemokines is CCR5, we speculated that CCL3/4/5–CCR5 axis could function between MSCs and CRC cells.

Next, we examined the expression of CCR5 in several CRC cell lines by flow cytometry, and found that these cells slightly expressed CCR5 on the cell surface (Fig. 1b, top). However, when cell membrane was permeabilized, a relatively strong CCR5 expression could be detected within the cytoplasm (Fig. 1b, bottom).

To investigate the functional consequences of the interaction between MSCs and CRC cells, we employed the co-inoculation mice model in which CRC cells (1 × 10^6^ cells) were mixed with BM-derived human MSCs (1 × 10^6^ cells) and injected subcutaneously into immunocompromised mice (Fig. 1c). The growth kinetics of the MSC-mixed tumors (CRC cells + MSCs) in mice were compared to those injected with CRC cells alone. We tested three CRC cell lines (i.e., HT29, SW480, and HCT116), and found that the mixture of MSCs significantly accelerated tumor growth in vivo in all cell lines (Fig. 1d). Mice injected with HT29 + MSCs displayed the significantly larger tumors compared to those injected with HT29 alone. Mice injected with SW480 + MSCs or HCT116 + MSCs displayed the visible tumors, whereas mice injected with SW480 or HCT116 alone did not exhibit tumor engraftment.

### CCR5-mediated cellular responses of CRC cell lines

**Fig. 2:**
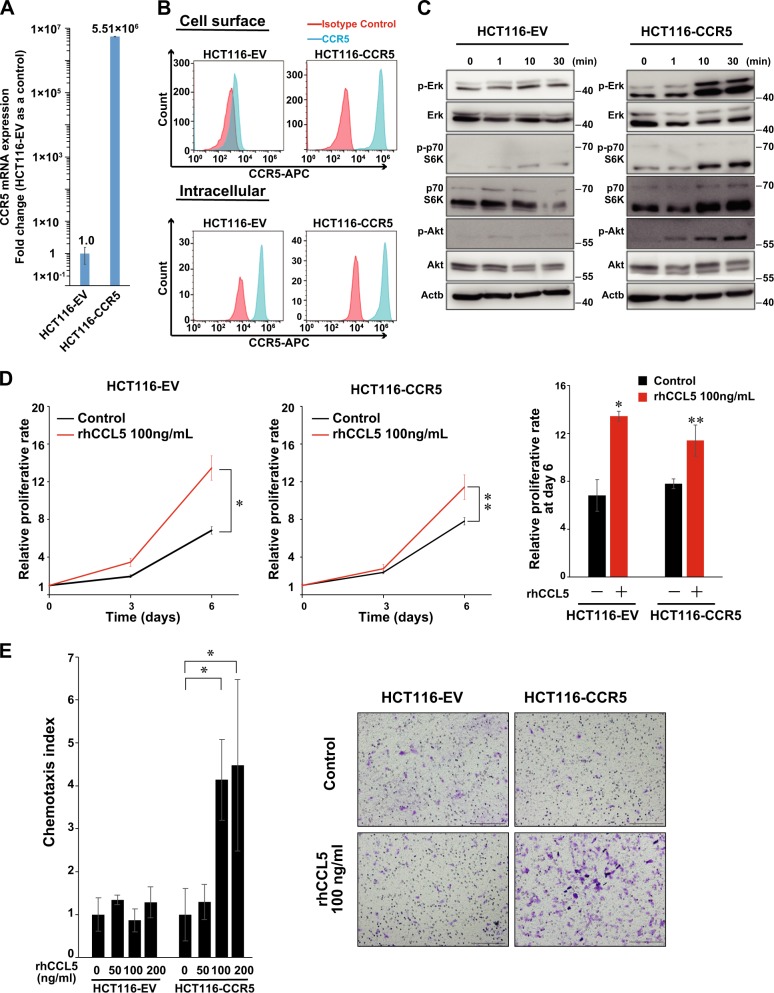
C-C chemokine receptor type 5 (CCR5)-mediated cellular responses in vitro. **a** CCR5 mRNA expression in HCT116 transfectant cells quantified by quantitative reverse transcription-PCR. Means; bar, ±SD, *n* = 3. **b** CCR5 cell surface expression (top) and intracellular expression (bottom) analyzed by flow cytometry. **c** Intracellular signaling of HCT116 transfectant cells treated with C-C motif chemokine ligand 5 (CCL5; 100 ng/ml). **d** Cell proliferation assay using Cell Counting Kit-8 assay. HCT116 transfectant cells treated with CCL5 (100 ng/ml) analyzed at day 0, 3, and 6. Normalize an absorbance at day 0 as 1. Mean; bars, ±SD, *n* = 3 (Student’s *t* test; *P = 0.008, **P = 0.03). **e** Chemotaxis index of HCT116 transfectant cells treated with various CCL5 concentrations (left). Mean; bars, ±SD, *n* = 6 (Student’s *t* test; *P < 0.001). Representative images of transwell membrane (right). Scale bar, 200 μm

To investigate the role of CCR5 in CRC progression, we established stable HCT116 transfected lines in which CCR5 or empty vector was introduced by retroviral transfection (referred as HCT116-CCR5 or HCT116-EV cells, respectively). We confirmed that the mRNA and protein levels of CCR5 were markedly higher in HCT116-CCR5 cells compared to the control cells (HCT116-EV cells) (Fig. 2a, b). To examine the intracellular signaling pathways via CCR5, we analyzed the phosphorylation of Erk, PI3K, and Akt after treatment with CCL5 (Fig. 2c). Upon exposure of HCT116-CCR5 cells to CCL5, there was notable phosphorylation of Erk, PI3K, and Akt. On the other hand, in HCT116-EV cells, CCL5 stimulation caused only the phosphorylation of PI3K, while it did not increase the phosphorylation of Erk and Akt. Next, to explore the CCR5-mediated cellular function, we analyzed the effect of CCL5 on cell proliferation, and found that CCL5 significantly enhanced the proliferation of both HCT116-EV and HCT116-CCR5 cells (Fig. 2d). The proliferation rates of HCT116-CCR5 cells with or without CCL5 were almost similar to those of HCT116-EV cells, indicating the overexpressed CCR5 did not confer any proliferative advantage. Furthermore, we performed an in vitro chemotaxis assay to investigate whether the chemokine axis via CCR5 could induce cell migration. As expected, CCL5 caused directional migration in HCT116-CCR5 cells, whereas it did not in HCT116-EV cells (Fig. 2e).

### MSC promotes CRC tumor growth in vivo via CCR5

**Fig. 3:**
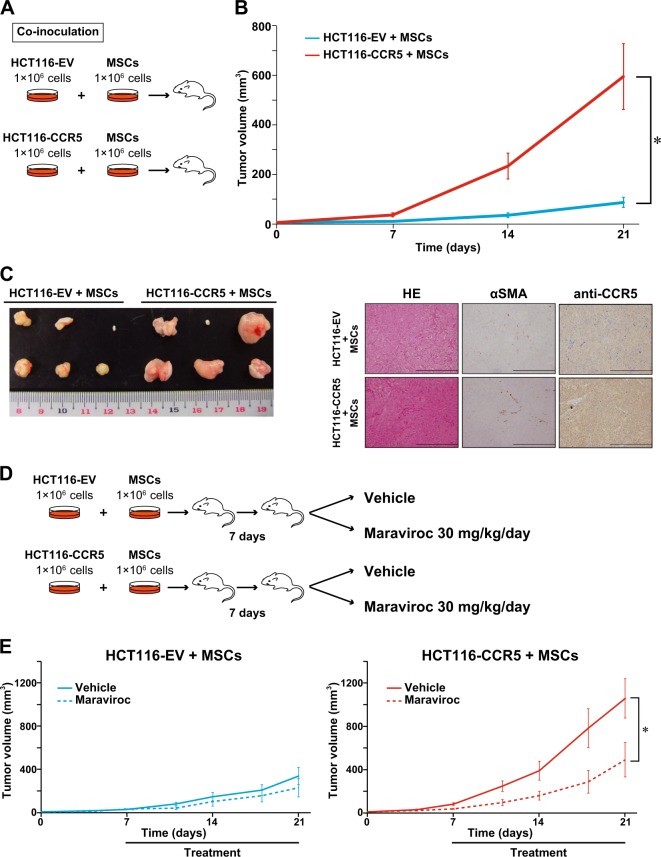
Enhancement of tumor-promoting effect of mesenchymal stem cells (MSCs) by C-C chemokine receptor type 5 (CCR5) axis. **a** Schema of co-inoculation mice model. HCT116 transfectant cells were mixed with MSCs. **b** Xenograft growth curves of HCT116-EV and HCT116-CCR5 with MSCs. Mean; bar, ±SE, *n* = 6 (Student’s *t* test; *P = 0.01). **c** Images of xenograft appearance at day 21 (left). Histological findings of xenograft (right). Hematoxylin and eosin (HE) and immunohistochemical staining for αSMA and anti-CCR5. Scale bar, 200 μm. **d** Schema of treatment schedule. **e** Xenograft growth curves of HCT116-EV (left) and HCT116-CCR5 (right) with MSCs. Dotted lines show treatment group with maraviroc 30 mg/kg/day, and solid lines show control group with vehicle. Means; bars, ± SE, *n* = 6 each group (Student‘s *t* test, *P = 0.042)

To assess the effect of CCR5 on the tumor growth in vivo, we inoculated HCT116-EV or HCT116-CCR5 cells (4 × 10^6^ cells) into immunocompromised mice and found that there was no significant difference in size between the two cells (Supplementary Fig. 1). Next, to examine the effect of MSC mixture, we employed the co-inoculation mice model in which HCT116-EV or HCT116-CCR5 cells (1 × 10^6^ cells) were mixed with MSCs (1 × 10^6^ cells), and then inoculated subcutaneously into the mice (Fig. 3a). The size of the tumors in mice injected with HCT116-CCR5 + MSCs was much larger compared to that in mice injected with HCT116-EV + MSCs (Fig. 3b, c). Three weeks after inoculations, we confirmed that CCR5 protein continued to be expressed in the HCT116-CCR5 + MSCs tumors, and that the pathological findings of the transplanted tumors were similar between the two cells (Fig. 3c). There were a few alpha-smooth muscle actin (α-SMA)-positive cells within these tumors.

To additionally verify the roles of CCR5, we attempted to suppress tumor growth with a CCR5 inhibitor, maraviroc. Seven days after inoculation, we started to inject maraviroc (30 mg/kg/day) repeatedly into the mice, and then compared the effect to the control vehicle up to 3 weeks after inoculation. In the HCT116-EV + MSCs tumors, there was no significant difference in size between the two groups (*P* = 0.37). On the other hand, in the HCT116-CCR5 + MSCs tumors, maraviroc significantly reduced tumor size (*P* = 0.042). These results suggest that CCR5 signaling is important for the interaction between CRC cells and MSCs, and that an inhibitor of CCR5 could suppress CRC progression.

### CCR5 expression in primary CRC and preoperative serum CCL3/4 levels are correlated with patients’ prognosis

**Fig. 4:**
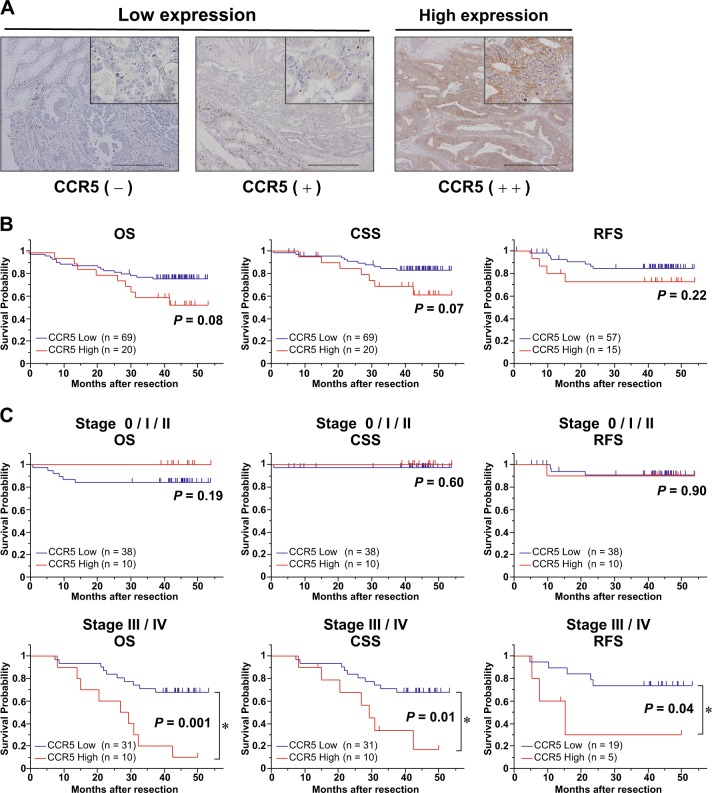
Correlation of C-C chemokine receptor type 5 (CCR5) expression in primary tumor with colorectal cancer (CRC) patients’ prognosis. **a** Immunohistochemical staining for CCR5 in primary CRC. Representative images are shown (scale bars, 500 μm). Magnified images were shown at right upper quadrant (scale bars, 50 μm). **b** Survival curves estimated by Kaplan-Meier method in all patients. Overall survival (OS: left), cancer-specific survival (CSS: middle), and recurrence-free survival (RFS: right) were shown. *P* value calculated by log-rank test. **c** Survival curves in subgroups divided into early stages (stage 0/I/II: top) and advanced stages (stage III/IV: bottom). *P* value calculated by log-rank test

To evaluate the clinical relevance of the abovementioned results, we examined CCR5 expression in CRC cells with 89 clinical specimens by immunohistochemical analysis. We found that 20 cases (20/89; 29%) had high levels of CCR5, whereas 69 cases (69/89; 71%) had comparatively lower levels (Fig. 4a). CCR5 expression was not associated with sex, age, tumor location, T-factor, N-factor, and M-factor (Table 1). To evaluate the effect of patients’ prognosis, we analyzed the overall survival (OS), cancer-specific survival (CSS), and relapse-free survival (RFS). Statistical analyses indicated that stages 0–IV patients with CCR5-high CRCs tended to exhibit shorter OS and CSS than those with CCR5-low CRCs (*P* = 0.08 and 0.07, respectively), although the difference was not significant (Fig. 4b). Furthermore, we performed subgroup analyses based on the stage-based classification, and found that stage III/IV patients with CCR5-high CRCs exhibited a significantly poorer prognosis (OS, CSS, and RFS) than those with CCR5-low CRCs (*P* = 0.001, 0.009, and 0.035, respectively; Fig. 4c). On the other hand, such a correlation was not observed in the analyses on stage 0/I/II patients. These results highlight the clinical importance of CCR5 expression in CRC, especially at advanced stages.

**Table 1:** Univariate analysis of patients and tumor characteristics with expression of CCR5

	CCR5		*P*
	Negative (*n* = 69)	Positive (*n* = 20)	
Age (mean)	68.4	69.3	0.64
Sex			0.61
Male	43	11	
Female	26	9	
Location			0.80
Colon	41	13	
Rectum	28	7	
UICC-TNM stage			0.80
0, I, II	38	10	
III, IV	31	10	
T-factor			0.59
Tis/T1/T2	24	5	
T3/T4	45	15	
N-factor			1.00
Negative	43	12	
Positive	26	8	
M-factor			0.76
Negative	55	15	
Positive	14	5	

*CCR5* C-C chemokine receptor type 5, *UICC*-*TNM* Union for International Cancer Control-TNM classification

**Fig. 5:**
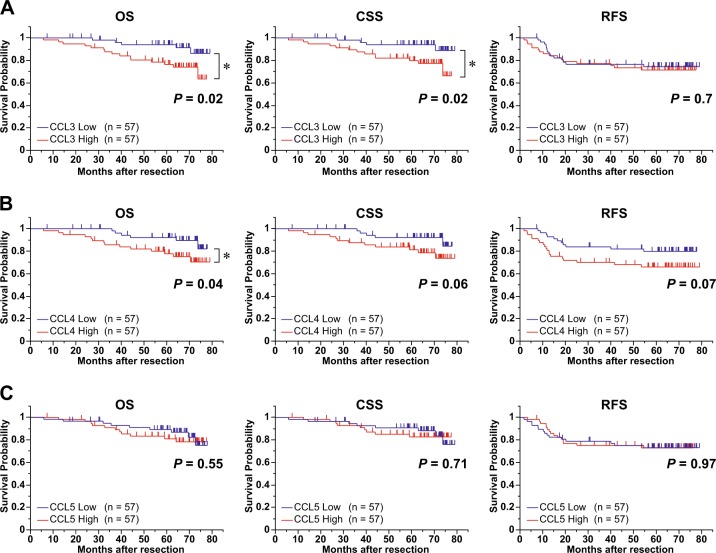
Correlation of preoperative serum levels of C-C motif chemokine ligand 3 (CCL3), C-C motif chemokine ligand 4 (CCL4), and C-C motif chemokine ligand 5 (CCL5) with colorectal cancer patients’ prognosis. **a**–**c** Survival curves of overall survival (OS), cancer-specific survival (CSS), and relapse-free survival (RFS) estimated by Kaplan-Meier method in CCL3 (**a**), CCL4 (**b**), and CCL5 (**c**). The median number of CCL3, CCL4, and CCL5 (i.e., 26.0, 21.0, and 24,900 pg/ml, respectively) was used as cutoff value. *P* value was calculated by log-rank test

Serum levels of CCL3, CCL4, and CCL5 have been recently reported to be useful as biomarkers of several cancers^16–21^. Therefore, we investigated whether they could be used as biomarkers of CRC progression. We measured the preoperative serum levels of CCL3, CCL4, and CCL5 from 114 CRC patients by enzyme-linked immunosorbent assay (ELISA) (Table 2). To evaluate the clinical outcome, we analyzed the OS, CSS, and RFS. Statistical analysis indicated that the cases with high CCL3 levels exhibited a significantly shorter OS and CSS compared to those with low CCL3 levels (*P* = 0.02 and 0.02, respectively), although such a correlation was not observed in RFS (Fig. 5a). The cases with high CCL4 levels exhibited a significantly shorter OS compared to those with low CCL4 levels (*P* = 0.04), and a similar correlation was also observed in CSS and RFS (*P* = 0.06 and 0.07, respectively) (Fig. 5b). On the other hand, there was no association between the CCL5 concentration and prognosis (Fig. 5c).

**Table 2:** Patients and tumor characteristics of analyzed serum sample from another cohort of 114 patients

Characteristics		No. of patients
Age, years		
Mean ± SD	65 ± 12.6	
Sex		
Male		56
Female		58
Location		
Colon		79
Rectum		35
Histology		
tub1/tub2		98
Others		16
T-factor		
Tis/T1/T2		35
T3/T4		79
N-factor		
Negative		76
Positive		38
M-factor		
Negative		95
Positive		19
UICC-TNM stage		
0, I, II		67
III, IV		47
CCL3, pg/ml		
<26.0		57
≥26.0		57
CCL4, pg/ml		
<21.0		57
≥21.0		57
CCL5, pg/ml		
<24,900		57
≥24,900		57

*SD* standard deviation, *CCL3* C-C motif chemokine ligand 3, *CCL4* C-C motif chemokine ligand 4, *CCL5* C-C motif chemokine ligand 5, *UICC-TNM* Union for International Cancer Control-TNM classification

## Discussion

MSCs have the multilineage differentiation potential and the capacity to home into the damaged tissues and modulate immune responses. Because of these properties, the therapeutic value of MSCs has been investigated in various diseases including regenerative medicine^5,22,23^. However, some studies have reported the risk of potential tumorigenicity related to the MSC-based therapy through genetic instability and transformation after prolonged cell culture^23–25^. Although there are not enough data/studies to draw a conclusion about the risk of tumorigenicity in the MSC-based therapy, the development of long-term follow-up in clinical settings is encouraged. Tumor-promoting effect of MSCs have been reported in various types of cancer, including CRC^26–30^. Recently, Chen et al. reported that CCL5 secreted by tumor necrosis factor-α-primed MSCs could promote tumor development via CCR1 expressed on CRC cells, which results in epithelial–mesenchymal transition via β-catenin/Slug pathway^29^. CCL5 is one of the C-C chemokines secreted from various cell types and interacts with CCR1, CCR3, and CCR5^31^. The CCL5–CCR5 axis has been reported to promote tumor progression by several lines of evidence^7,32–35^. Karnoub et al. showed the essential role played by CCL5–CCR5 axis in breast cancer metastasis to lungs^7^. Velasco-Velazquez et al. showed that CCL5–CCR5 axis was preferentially activated in more malignant subtype of breast cancer, and that a CCR5 inhibitor, maraviroc, reduced the progression of CCR5^+^ breast cancer cells in vitro and in vivo^35^. In CRC, one report showed that CCL5/CCR5 expression was upregulated in primary and metastatic CRC^36^, whereas another report showed that low CCR5 expression was correlated with advanced stages and reduced CD8^+^ T-cell infiltration^37^, indicating that the role of CCR5 in CRC is still controversial. Recently, Halama et al. reported that CCL5 produced by T lymphocytes in CRC liver metastases has tumor-promoting effects on tumor cells and tumor-associated macrophages (TAMs), and that the CCR5 inhibitor, maraviroc, led to tumor reduction through repolarization of TAMs^38^. CCL3–CCR5 axis has pro-tumorigenic effects on oral squamous cell carcinoma^39^, and lung metastasis^40^. Tanabe et al. reported that cancer-associated fibroblasts (CAFs) accumulated into tumor sites via CCL3–CCR5 axis, and that CCR5 blockage with maraviroc could suppress tumor growth in a mouse colitis-associated CRC model^41^. Sasaki et al. reported that CCL4–CCR5 axis could contribute to bone metastasis of breast cancer; cancer cell-derived CCL4 could induce CCR5-exrpressing fibroblasts to support tumor progression^42^.

CCR5 is expressed in several types of cancer cells as well as immune cells, T lymphocytes, dendritic cells, leukocytes, and stromal cells^43^. Only a few studies have investigated the relationship between CCR5 expression and patients’ prognosis^44–47^. In the present study, CCR5 expression was correlated with poor prognosis of CRC patients, especially those at more advanced stages (stage III/IV) (Fig. 4c). Thus, we speculate that the chemokine axis via CCR5 might be one of the critical axes for tumor progression in advanced stages.

CAFs have been known to contribute to cancer progression^48–50^. Resident fibroblasts, smooth muscle cells, endothelial cells, and epithelial cells are assumed to be the sources of CAFs in the TME. Recently, some researchers reported that MSC is a potent source of CAFs. For example, Worthley et al. reported that CAFs in gastric cancer and rectal cancer of female patients who received BM transplants from male donors were positive for Y-chromosome marker derived from a male BM donor, which suggests that the CAFs are derived from BM^13^. Using mouse models of inflammation-induced gastric dysplasia, Quante et al. showed that at least 20% of CAFs were originated from MSCs present in the BM, and that CAFs were recruited to the tumor in a transforming growth factor-β-dependent manner together with the CXCL12–CXCR4 axis^14^. Jung et al. reported that CXCL16 secreted from prostate cancer facilitates conversion of MSCs into CAFs^11^. In the present study, we found that scattered spindle-shaped cells like fibroblasts in the xenograft co-inoculated with MSC, and that a few of them were αSMA-positive (Fig. 3c), which might delineate that some of co-inoculated MSCs could differentiate to αSMA-positive stromal cells like CAFs. Although MSCs have important roles in the development of TME, their mechanism of action remains to be elucidated.

As for the serum CCR5 ligands (i.e., CCL3, CCL4, and CCL5), serum levels of CCL3 and CCL4 were significantly correlated with patients’ OS in diffuse large B-cell lymphoma^16^. In chronic lymphocytic leukemia, serum levels of CCL3 and CCL4 were elevated^17^, and high CCL3 levels could be a predictor of poorer prognosis^18^. In oral squamous cell carcinoma, high serum CCL4 levels were associated with a more advanced stage^19^. High serum CCL4 levels were also reported to be associated with a poor OS in lung adenocarcinoma^20^. Recently, serum levels of CCL4 and CCL5 in patients with liver cirrhosis were reported to be sensitive predictors for the presence of hepatocellular carcinoma^21^. To our knowledge, the association of CRC with serum levels of CCL3, CCL4, and CCL5 has not been reported so far. Thus, our study is the first to evaluate this association.

The human immunodeficiency virus (HIV) utilizes CCR5 as a co-receptor with CD4 to enter target cells; therefore, the CCR5 has been heavily evaluated as an important treatment target of HIV-1 infection^43^. Repurposing the drugs already approved as non-anticancer drugs is an attractive strategy because of the cost, safety, and clinical validation of protocols^51^. In this context, CCR5 is one of the attractive targets for cancers, because it has been already used as a target for inhibition of HIV entry^52,53^. Maraviroc is approved by U.S. Food and Drug Administration, and commonly used for treatment of HIV infection. A new CCR5 antagonist in advanced clinical trials for treatment of HIV infection is cenicriviroc (TAK-652), which is a second-generation small-molecule CCR5 antagonist and dual chemokine receptors inhibitor for CCR2 and CCR5^54^. Development of another dual chemokine-based agent (CCR5/CXCR4) is in now ongoing^53^. As for cancer treatments, various CCR5 antagonists, including maraviroc, vicriviroc, TAK-779, Met-CCL5, and anibamine, have been shown to have antitumor effect in preclinical mouse models^34^. Among them, maraviroc has been most intensively investigated in various mouse models.

In this report, we have shown that CCL3/4/5–CCR5 axis facilitates tumor progression by the interaction between MSCs and CRC cells. These results suggested that some chemokines secreted from MSCs could be important factors among the interaction between MSCs and cancer cells in the TME. Our findings that α-CCR5 treatment blocked the MSC-induced tumor progression suggest that the inhibition of MSC–CRC interaction could represent an effective treatment strategy for CRC and underscore the need for clinical trials of these drugs.

## Materials and methods

### Cell lines and reagents

SW480, SW620, HT29, HCT116, and DLD1 human CRC cells were supplied from American Type Culture Collection and were maintained in low-glucose Dulbecco’s modified Eagle’s medium (DMEM; Nacalai Tesque, Kyoto, Japan) containing 10% fetal bovine serum and 1% penicillin/streptomycin mixture. Human BM-derived MSCs were supplied from Lonza (PT-2501; Basel, Switzerland) and cultured with MSCGM BulletKit (PT-3001; Lonza). We used them for experiments by passage 5 according to the manufacturer’s protocol. Recombinant human CCL5/RANTES protein was obtained by R&D systems (Minneapolis, MN, USA). An antagonist of the CCR5, maraviroc, was purchased from Cayman Chemical (Ann Arbor, MI, USA).

### Chemokine array

Human chemokine antibody array was obtained from RayBiotech (Norcross, GA, USA). After MSCs were cultured for 48 h, the supernatant was collected and then analyzed by human chemokine antibody array kit (RayBiotech), according to the manufacturer’s protocol. The signal intensity of each chemokine spot was measured by a LAS-3000 mini lumino image analyzer (Fjifilms, Tokyo, Japan). After substrate negative control value, relative pixel density value was calculated with the positive control as 100.

### Flow cytometry

CRC cells at subconfluency (50–70%) were detached with 2 mM EDTA in phosphate-buffered saline (PBS). After washes, 5 × 10^5^ CRC cells were incubated on ice for 30 min with anti-human CCR5 antibody (monoclonal mouse IgG2b clone #45531; R&D Systems). After washing, they were incubated with allophycocyanin-conjugated anti-mouse secondary antibodies (Cat # 405308, BioLgend, San Diego, CA, USA) and analyzed on a BD Accuri C6 flow cytometer (BD Biosciences, San Jose, CA, USA). To evaluate CCR5 intracellular expression, cells were permalized with BD Cytofix/Cytoperm Fixation/Permeabilization Kit (BD Biosciences) according to the manufacturer’s protocol.

### Retroviral transduction of HCT116 human colon cancer cell

Human CCR5 cDNA was obtained from peripheral blood mononuclear cell of healthy donor and amplified by RT-PCR. The following primers were used, with each containing either a *Not*I or *Bam*HI site. 5′-CCGGCGGCCGCGCCACCATGGATTATCAAGTGTCAAG-3′ and 5′-CCGGGATCCTCACAAGCCCACAGATATTT-3′. The obtained PCR fragment was digested with *Not*I and *Bam*HI, and subcloned into retroviral vector pHIV-ZsGreen (from Hisamori S, Kyoto University). The resulting vector was confirmed by sequencing to coincident with the published data (GenBank Accession No. NM_000579). Using lentiviral packaging plasmid psPAX2 and VSV-G envelope expressing plasmid pMD2.G, HCT116 CRC cells were transfected pHIV-CCR5-IRES-ZsGreen with produced lentivirus. After initial transfection, the high expressed ZsGreen cells were isolated using BD FACSAria II cell sorter (BD Biosciences).

### qRT-PCR analyses

Total RNA was extracted and reverse transcription was performed with RevaTra Ace (TYOBO, Osaka, Japan) according to the manufacturer’s protocol. The synthesized cDNA was quantified with StepOnePlus Real-Time PCR system (Thermo Fisher Scientific, Waltham, MA, USA) and THUNDERBIRD SYBR qPCR Mix (TOYOBO). The flowing primers were used: CCR5, 5′-CACAGGGCTGTGAGGCTTAT-3′ and 5′-TCACCTGCATAGCTTGGTCC-3′; ACTb, 5′-GCAAAGACCTGTACGCCAAC-3′ and 5′-ACATCTGCTGGAAGGTGGAC-3′.

### Western blot analysis

Cells were lysed with SDS sample buffer (70 mM Tris-HCl, 3% SDS, and 10% glycerol) with inhibitor cocktails of protease and phosphatase. Primary antibodies were rabbit monoclonal anti-phospho-p44/42 kinase (Thr202/Tyr204: Cell Signaling Technology, Danvers, MA, USA), anti-p44/42 kinase (Cell Signaling Technology), rabbit monoclonal anti-phospho-Akt (Ser473:Cell Signaling Technology), rabbit monoclonal anti-Akt (Cell Signaling Technology), rabbit polyclonal anti-phospho-p70 S6 kinase (Thr389: Cell Signaling Technology), rabbit polyclonal anti-p70 S6 kinase (Cell Signaling Technology), and mouse monoclonal anti-β-actin-peroxidase (Sigma-Aldrich, St. Louis, MO, USA). For stimulation experiments, cells were starved for 24 h with serum-free DMEM and then stimulated with 100 ng/ml of CCL5. Cell lysates were obtained after the indicated times stimulation and subjected to SDS-polyacrylamide gel electrophoresis, immunoblotted with the primary antibodies followed by horseradish peroxidase-conjugated secondary antibodies, and then analyzed by a lumino image analyzer.

### Cell proliferation assay

Cell proliferation was assayed with Cell Counting Kit-8 (Dojin, Kumamoto, Japan) according to the manufacturer’s protocol. Cells were cultured at a density of 2000 cells/well of 96-well plate in serum-free media with or without chemokine ligand CCL5. Each sample were analyzed absorbance at day 0, 3, and 6, and calculated relative rate with day 0 value as 1.

### Chemotaxis assay

Migration assay was assayed in 24-well Transwell cell culture chambers (8 μm-pore membrane) (Corning, NY, USA). Membrane were precoated with 5 μg of fibronectin in a volume of 50 μl on the lower surface, dried, and washed before use. After 24 h starvation with serum-free DMEM, HCT116-EV or CCR5-OE cells (2 × 10^4^ cells/well) were added to the upper chamber and serum-free media with indicated ligand concentration was added to the lower chamber. After 6 h incubation, cells attached on the lower surface of the membrane were counted in at least five different fields (original magnification × 200).

### Animal tumor model

Eight- to 11-week-old female KSN/slc nude mice (Japan SLC, Hamamatsu, Japan) were bred under specific pathogen-free conditions. Protocols of animal assay were approved by the Animal Experiment Committee of the University of Kyoto. For assessment of the role of coexisted MSC, the mice were divided into two group, and 1 × 10^6^ CRC cells alone or both 1 × 10^6^ CRC cells and 1 × 10^6^ MSCs in 100 μl PBS were subcutaneously injected into the flanks of each mouse. To examine maraviroc inhibitory effect on interaction between CRC and MSCs, HCT116-EV and HCT116-CCR5 cells were co-inoculated with MSCs in the same manner and the mice were randomized in control group (vehicle, 100 μl PBS with 5% dimethyl sulfoxide, intraperitoneal (i.p.)) and treatment group (maraviroc 30 mg/kg, i.p.). Treatment was started at day 7 after cell inoculation and continued for 14 days. Tumor volumes were calculated using the formula (length × width^2^) × 0.5.

### Immunohistochemistry

Formalin-fixed, paraffin-embedded sections were stained with anti-human CCR5 antibody (anti-human CCR5 antibody: monoclonal mouse IgG2b clone # 45523, dilution 1:50) (R&D) by the avidin-biotin immunoperoxidase method. Antigen retrieval was conducted in citrate buffer (pH: 6.0) at sub-boiling temperature for 10 min. The primary antibodies were applied and incubated over night at 4 °C. For primary CRC tissue, the expression of CCR5 was classified into three groups based on the expression intensity: ­−, absent; +, positive (almost cytoplasmic staining); ++, strong positive (membranous staining) and was defined to be high expression if the tumors possessed strong positively stained (++) cells and to be low expression if positively stained or absent. The slides were evaluated without prior knowledge of other data.

### Enzyme-linked immunosorbent assay

Serum protein levels of CCL3, CCL4, and CCL5 were measured with Human CCL3, CCL4, and CCL5 DuoSet ELISA (R&D), according to the manufacturer’s protocol. The serum protein levels of CCL3, CCL4, and CCL5 were divided into high group and low group. Median vale was used as cutoff value.

### Patients and clinicopathological data

A total 89 of resected CRC samples were collected at Kyoto University Hospital in 2008. For the analysis of serum CCL3, CCL4, and CCL5 levels, preoperative serum samples were collected from 114 CRC patients between 2011 and 2014. These study protocols were approved by the Institutional Review Board of Kyoto University, and the patients provided their consents for data analysis.

### Statistical analysis

Analyzed values were expressed as means ± standard deviation in in vitro experiments and means ± standard error in in vivo experiments. Categorical data were determined with Fisher’s exact test. Continuous variables were determined with Student’s *t* test. The log-rank test was used for analysis of OS, CSS, and RFS. All analyses were two-sided, and *P* value < 0.05 was considered as statistically significant. Statistical analyses were performed using JMP Pro software, version 14.0.0 (SAS Institute Inc, Cary, NC, USA).

## Supplementary information

Supplementary Figure 1
