# Supplementary material for: Bone marrow-derived mesenchymal stem cells promote colorectal cancer progression via CCR5
Source: Cell Death Dis. 2019 Mar 19;10(4):264. doi: 10.1038/s41419-019-1508-2 (PMC6424976; doi:10.1038/s41419-019-1508-2)
Supplement: Supplementary file 1 — Supplementary Figure 1 [file 41419_2019_1508_MOESM1_ESM.pdf]

Figure S1

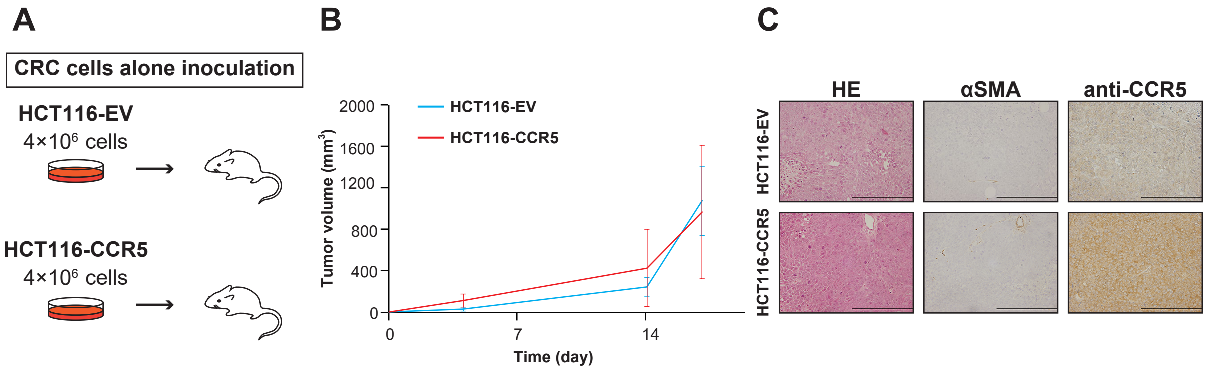

Supplementary Figure S1. Effect of CCR5 on the tumor growth in vivo.

A Schema of inoculation mice model. HCT116 transfectant cells were inoculated into mice. **B** Xenograft growth curves of HCT116-EV and HCT116-CCR5. Mean; bar, ± SE, n=6. **C** Histological findings of xenograft. H&E and immunohistochemical staining for aSMA and anti-CCR5. Scale bar, 200 μm.
